# Psychosis of Epilepsy: An Update on Clinical Classification and Mechanism

**DOI:** 10.3390/biom15010056

**Published:** 2025-01-03

**Authors:** Zhiruo Qiu, Jiahui Guo, Bofei Chen, Jiajia Fang

**Affiliations:** Department of Neurology, The Fourth Affiliated Hospital, International Institutes of Medicine, Zhejiang University School of Medicine, Yiwu 322000, China; 22318457@zju.edu.cn (Z.Q.); 22318451@zju.edu.cn (J.G.); 22318450@zju.edu.cn (B.C.)

**Keywords:** psychosis of epilepsy, mechanism, biomarkers, neuroimaging, neurobiology, genetics

## Abstract

Epilepsy is a prevalent chronic neurological disorder that can significantly impact patients’ lives. The incidence and risk of psychosis in individuals with epilepsy are notably higher than in the general population, adversely affecting both the management and rehabilitation of epilepsy and further diminishing patients’ quality of life. This review provides an overview of the classification and clinical features of psychosis of epilepsy, with the aim of offering insights and references for the clinical diagnosis and treatment of various types of psychosis of epilepsy. Additionally, we examine the potential pathophysiological mechanisms underlying the psychosis of epilepsy from three perspectives: neuroimaging, neurobiology, and genetics. The alterations in brain structure and function, neurotransmitters, neuroinflammatory mediators, and genetic factors discussed in this review may offer insights into the onset and progression of psychotic symptoms in epilepsy patients and are anticipated to inform the identification of novel therapeutic targets in the future.

## 1. Introduction

Epilepsy is a chronic, noncommunicable disease of the brain that affects people of all ages. According to World Health Organization estimates, epilepsy is one of the most common neurological diseases, affecting around 50 million people of all ages around the world [1]. Epilepsy alone may have a serious negative impact on individuals, families, and society. To make matters worse, psychiatric comorbidity of epilepsy is very common, with an incidence of more than 50% [2,3]. In the medical literature, the psychotic disorder that occurs in people with epilepsy is often referred to as psychosis of epilepsy (POE). This is a term applied to a group of mental disorders with a unique phenomenology and pathogenesis that may be closely related to seizures [4]. It is worth noting that POE here refers specifically to psychotic symptoms, that is, one or more of the following four categories: delusions, hallucinations, grossly disorganized or catatonic behavior, and negative symptoms that progress with preserved consciousness [Diagnostic and Statistical Manual of Mental Disorders (Fifth Edition)/DSM-V] [5]. It does not include other mental disorders such as depression, bipolar disorder, obsessive-compulsive disorder, and so on. A systematic review of 58 valid articles showed that patients with epilepsy had a pooled odds ratio for risk of 7.8 for developing psychosis compared with controls, and the pooled prevalence was estimated to be 5.6% (95%CI: 4.8–6.4%). In addition, the prevalence of psychotic disorders in temporal lobe epilepsy (TLE) is even higher, with an estimated 7% (95% CI: 4.9–9.1%) [6], whereas the probability of psychotic disorders in extra-TLE is about 4.3% [7]. POE not only affects the treatment and rehabilitation of epilepsy but also greatly reduces the quality of patients’ lives.

A century ago, however, psychiatry and neurology were considered to be distinct disease spectra: neurology considered disorders of the nervous system to have a definite cause and clear anatomic pathology, while psychiatry studied psychological disorders without clear pathology, that is, idiopathic functional disorders [8]. Until later, more and more studies found that the prevalence of psychotic disorders in patients with epilepsy was higher than that in the general population [6]. What is more, it has been shown that the presence of psychosis increased the risk of subsequent seizures and that the presence of epilepsy also increased the risk of subsequent psychotic disorders [9]. Therefore, the relationship between the two is complex and cannot be described by simple causation. There may be complex interactions under the influence of genetics, structural pathological changes, and environment; that is, epilepsy and psychosis are bidirectional. Although this issue is still inconclusive, many scholars have made some discoveries in the aspects of neuroimaging, neurobiology, and genetics of POE and proposed certain possible mechanisms that need to be further studied and verified.

This review will center around the clinical classification and possible pathophysiological mechanisms of POE, with a focus on the mechanisms, and will elaborate on the possible relevant mechanisms from the perspectives of neuroimaging, neurobiology, and genetics. The aim of this review is to provide some clues for the occurrence and development of psychotic symptoms in patients with epilepsy and hope to provide some reference for finding new therapeutic targets in the future.

## 2. Classification and Clinical Features

POE refers to a concomitant psychotic disorder or syndrome with psychotic symptoms as the main clinical manifestation in patients with epilepsy. Traditionally, according to their temporal relationship, they are divided into ictal, interictal, and postictal POEs. Some patients may exhibit psychotic symptoms after seizure control, which is called forced normalization [4,10]. In addition to the above four categories, some scholars have also proposed preictal POE [11]. Because any psychotic symptoms can be present in POE, it is often difficult to symptomatically distinguish psychotic symptoms of epilepsy from other psychotic disorders. In general, however, psychosis develops later in patients with epilepsy (mean age, 30.1 years) than in patients with primary schizophrenia (mean age, 26.6 years) [12]. The following will briefly introduce the diagnostic criteria and clinical features of the five POEs mentioned above.

### 2.1. Preictal Psychosis of Epilepsy (PrP)

The preictal psychosis of epilepsy (PrP) is less common than interictal and postictal POE. It has been proposed that it usually appears within a few hours (rarely up to 3 days) before the onset of seizures and that the psychotic symptoms are usually not specific [11]. These symptoms include loss of reality and depersonalization experiences, obsessive thinking, premonitory signs of ideological movement, hallucinations, anxiety, euphoria, and so on. They usually disappear with the seizure but are not associated with detectable electroencephalogram (EEG) changes [11]. It is crucial to differentiate between the psychotic symptoms associated with PrP and the premonitory symptoms. The primary distinction lies in the alterations observed in EEG readings. Premonitory symptoms are frequently characterized by abnormal discharges on the EEG, whereas the psychotic symptoms of PrP do not induce such changes. In addition, premonitory symptoms are typically followed by seizures within approximately thirty minutes, whereas there are often several hours to a few days between the onset of psychotic symptoms and the occurrence of seizures in PrP. Currently, research on PrP remains limited, and it is seldom addressed by scholars in discussions of POE. Future investigations into the symptomatology and EEG characteristics of PrP are essential. Additionally, we propose that depth-recording studies hold significant value in distinguishing between premonitory symptoms and those associated with PrP.

### 2.2. Ictal Psychosis of Epilepsy (IP)

Ictal psychosis of epilepsy (IP) is a non-convulsive epileptic state in the form of psychotic symptoms that occur primarily in the temporal lobe and rarely outside the temporal lobe (i.e., the frontal lobe) [11]. Psychomotor states of temporal lobe origin may be characterized by thoughts of persecution, anxiety/fear, negativity, irritability, or aggression [13]. Hallucinatory symptoms, depersonalization, or derealization that occur during seizures are almost always associated with other symptoms (automaticity, altered consciousness) of seizures that occur in the temporal or frontal lobes. Therefore, for any sudden onset of mental abnormalities, an EEG examination is very necessary [10]. It has been shown that medio-basal focal spike activity has a significant advantage in TLE patients with IP, thus suggesting an association between deep temporal and adjacent limbic system dysfunction and IP [14]. In addition, in a patient with IP, which occurs in the frontal lobe, the EEG showed about 3 Hz sharp waves predominantly in the left frontal area [15]. Since IP is a manifestation of seizures, it can be controlled by effective epilepsy treatment without the need for antipsychotic drugs (APDs).

### 2.3. Interictal Psychosis of Epilepsy (IIP)

For interictal psychosis of epilepsy (IIP), some scholars define it as a comorbid psychotic disorder that is often of prolonged duration and does not have a clear temporal relationship with seizures [11]. According to a conventional definition, IIP was determined as (1) the development of psychosis after the onset of epilepsy, (2) the occurrence of psychosis without distinct antecedent seizures, and (3) psychosis lasting more than 24 h in a state of clear consciousness [16]. Although there is no consensus on the definition, most scholars have a common view that IIP occurs after the onset of epilepsy, appears during the interictal period, and has no clear time relationship with the onset of epilepsy. One study found that among 1434 patients with epilepsy, the prevalence of IIP was 2.2%, while the prevalence of postictal psychosis was 3.7%. It has been found that IIP patients show more conceptual disturbances (the destruction of the purpose and coherence of thinking, such as tautology, digression, and loose association) and negative symptoms (the decline or absence of mental function, mainly manifested as emotional dullness or withdrawal and lack of interest) and that the distribution of seizures caused by temporal or extratemporal structures is almost equal [17]. There is no consensus on whether IIP requires treatment of antipsychotic symptoms. Data from a multicenter, consecutive cohort of 320 psychotic episodes in 155 IIP patients showed that most episodes lasted from months to years, and the average duration was 82.7 weeks. Moreover, the duration of psychotic symptoms was shorter when APDs were started early in the course of treatment [18]. This finding is consistent with previous findings in first-episode psychosis (FEP) [19]. Therefore, if it is found in clinical practice that patients with epilepsy develop definite interictal psychotic symptoms, early initiation of treatment with APDs may be more beneficial to improve symptoms. Although some differences in efficacy have been reported, there is no conclusive evidence that second-generation APDs are more effective than first-generation APDs. Expert opinion is that APDs should be selected based on efficacy and tolerability [20]. A case–control study found that patients with IIP had a higher frequency of seizures, especially focal epilepsy, than the epilepsy control group. Moreover, a higher frequency of seizures in patients with focal epilepsy was related to the first occurrence of IIP, while no correlation was found in the whole group and in generalized epilepsy patients [21]. Therefore, seizure control is also an important treatment principle for the prevention and treatment of IIP, especially for focal epilepsy. While IIP occurs in patients with generalized epilepsy, more non-epileptic risk factors should be considered. In addition, epilepsy surgery may also have a certain impact on IIP. A 1-year follow-up of 12 patients with chronic interictal psychosis combined with intractable epilepsy showed that four patients worsened at 6 months and 1 year after treatment, two patients deteriorated at 6 months and improved at 1 year, three remained unchanged, and three improved [22]. Although the effect of epilepsy surgery on psychotic symptoms in patients with IIP is not clear at present, in general, many scholars believe that epilepsy surgery has a positive effect on the quality of life of POE patients, and epilepsy surgery can be performed under close psychiatric supervision [22,23,24].

### 2.4. Postictal Psychosis of Epilepsy (PIP)

Postictal psychosis of epilepsy (PIP) is the most common POE and represents about 25% of all POE [4,11]. PIP, as the name suggests, refers to psychotic symptoms that occur after an epileptic seizure. In fact, however, the psychotic symptoms do not occur immediately after the seizure; instead, the lucid interval (i.e., a period of normal mental state from the end of a seizure to the beginning of an onset of psychosis) is a typical feature of most patients and lasts an average of 1–6 days [25,26]. There is no consensus on the duration of the lucid interval. Other scholars have suggested that the lucid interval can be 2.3–72 h [27], 8–72 h [28] or 12–72 h [29]. In any case, many studies have found and agreed on the existence of a lucid interval and proposed that psychotic symptoms of PIP would appear within a few hours to a few days after the seizure, generally no more than 1 week. Reports suggest that PIP may be characterized by relatively prominent emotionally charged delusions and hallucinations [28,30]. Violent or suicidal behavior may be a specific marker of PIP [17,31,32]. Analysis of EEG data confirmed a significant correlation between TLE and PIP, and the prevalence of PIP in TLE was about 7.0% [17,31]. These psychotic symptoms usually subside spontaneously within a month and may not require the use of APDs [25]. However, for frequent episodes of psychosis, the use of APDs should be considered. Sedative medications are often required to discourage inappropriate social behavior and to terminate or mitigate PIP symptoms, both during the initial phase of PIP and during episodes of PIP. Regarding the choice of medication, some experts recommend the use of benzodiazepines, while others prefer the combination of benzodiazepines and APDs. No matter which agent is chosen, effective sedation is of the utmost importance [33].

### 2.5. Forced Normalization (FN)

The concept of forced normalization (FN) was first introduced in 1953 by Heinrich Landolt and referred to the disappearance of epileptiform activity in the EEG of patients with drug-resistant epilepsy accompanied by acute behavioral deterioration. Later, Trenbach shifted the focus of this concept developed by Landolt to a clinical perspective, calling it “alternative psychoses” and proposing that epilepsy in FN patients did not actually disappear. In 1991, Wolf coined the term “paradoxical normalization,” in which EEG and epileptic activity returned to normal but actually continued to deteriorate clinically. He thought that epilepsy was still active in the subcortex, but the epileptic discharges may lead to psychosis along a different pathway [34].

Since Landolt’s early observations, many patients with FN have been described, making the existence of this phenomenon no longer suspect. In 1999, Krishnamoorthy et al. proposed recommended diagnostic criteria for FN, including a definitive diagnosis of epilepsy, the presence of acute/subacute behavioral disorders, reduced EEG seizure activity, and/or complete cessation of clinical seizures for at least 1 week [35]. Its clinical manifestations are usually thought disorders, delusions, hallucinations, marked mood changes, hypomania or depression, and anxiety with depersonalization and derealization [35]. It is worth noting that almost all the cases reported by Landolt were caused by anti-seizure medications (ASMs). It has been found that ASMs, such as phenytoin, carbamazepine, lamotrigine, and lacosamide [36,37], may cause FN. A review found that FN was mainly caused by the use of ASMs (48.5%), epilepsy surgery (31.8%), or vagus nerve stimulation (VNS) (13.6%). A total of 87% of FN patients by ASM triggered with withdrawal presented a complete resolution of symptoms in comparison to 28.5% of patients triggered by surgery [34]. In addition, FN may also be related to the neurobiological mechanism after seizure control. The current conjecture on the mechanism of FN may be related to electrical and pharmacological kindling phenomena [38], patterns of altered functional connectivity in the TLE [39], and gray matter structural abnormalities in the limbic system related to psychiatric disorders [40]. The phenomenon of electrical kindling was defined by Goddard et al., who proposed that a model of epilepsy could be induced by low-amplitude and high-frequency electrical stimulation of susceptible brain regions, such as the hippocampus, etc. Pharmacological kindling refers to the effect of drugs (such as amphetamine, cocaine, and lidocaine) on the limbic system of rodents to produce psychotic-like symptoms, which is mainly regulated by the dopaminergic pathway [41]. Wahnschaffe et al. found that injection of the dopamine D2 agonist LY 171,555 into the ipsilateral amygdala ignited rats and produced anticonvulsant effects [42]. Similarly, it has been proposed that the hypersensitization of dopaminergic receptors by cocaine and apomorphine inhibits the progression of the amygdala to generalized seizures and promotes this progression by blocking these receptors with antineuroleptic agents such as haloperidol [43]. These two antagonistic kindling mechanisms partly explain why psychotic symptoms occur after seizure cessation, providing a theoretical basis for FN. However, the exact neurobiological mechanism behind this phenomenon is still unclear, and it is expected to be further studied in the future. In terms of the treatment of FN patients, the current data suggest that the use of APDs does not seem to play a decisive role in the rehabilitation of psychosis, while the discontinuation of the ASMs has a significant effect on the relief or disappearance of psychotic symptoms [34] (Table 1).

## 3. The Effects of ASMs

ASMs are generally the first choice for the treatment of epilepsy. Some studies showed psychoses after some ASM administrations. The relationship between ASMs and FN is very close, and the specific content has been described in the section related to FN, which will not be repeated here. The following content mainly focuses on the influence of ASMs on IIP and PIP. Naoto Adachi et al. studied 2067 patients with focal epilepsy for every six-month period after administration of a new drug (either ASMs or non-ASMs) between 1981 and 2015. During the first six months, 89 patients had 105 psychotic episodes (24 PIP and 81 IIP) [44]. The incidence of psychosis was significantly increased with the use of second-generation ASMs, particularly topiramate (5.8%) and lamotrigine (3.7%). Whereas the daily dosage of ASMs was not significantly associated with the incidence of psychosis [44]. Although previous studies have shown that topiramate is associated with a high risk of psychosis [45], other scholars have found different results [46]. Moreover, some studies have found that the risk of psychosis with lamotrigine appears to be low [46,47]. These differences leave it uncertain whether specific ASMs have a prominent role in the development of psychotic disorders in patients with epilepsy, and it is necessary to increase the sample size to draw strong conclusions. Anyway, paying attention to psychotic symptoms in epilepsy patients at the beginning of drug use is necessary, especially in second-generation ASMs. What is more, a history of previous psychotic disorder and a familial predisposition are vital risk factors that should always be considered when selecting the appropriate ASMs [48].

## 4. Pathogenic Mechanism of POE

The pathogenic mechanism of POE has not been conclusively established, but a number of studies have investigated its neuroimaging, neurobiology, and genetic mechanisms. The following will introduce the research status of POE in the above three aspects in detail.

### 4.1. Neuroimaging

In neuroimaging, there have been studies from traditional structural magnetic resonance imaging to functional neuroimaging, but the results of different studies are not completely consistent. It is possible that the inconsistent results are the result of inadequate sample sizes or less advanced techniques. In any case, it would still be of interest to further expand the sample size/use the latest techniques to investigate POE neuroimaging in the future.

#### 4.1.1. Structural Neuroimaging

With the development of neuroimaging technology, it has become more and more important in the diagnosis and treatment of diseases. It is well known that lesions in the medial temporal lobe are closely related to epilepsy, so early studies on brain structure in POE have also focused on the hippocampus and amygdala. These findings varied, including reduced bilateral hippocampal and amygdala volumes [49], no difference in total hippocampal volume [50,51], reduced left hippocampal volume [52], and increased bilateral amygdala volume [51]. There are different sub-regions in the hippocampus, and each sub-region may have different functions and neural pathways [53]. Therefore, studies of hippocampal subregions may be more informative. A large cohort of 50 patients with POE and 50 patients with epilepsy examined the volume of the total hippocampus and each subregion and found that the volumes of the posterior hippocampus were reduced bilaterally in POE patients. In addition, volume loss was observed on the posteroanterior gradient, with a severe reduction in the hippocampal tail, a moderate reduction in the body, and no difference in the head [54]. However, a study the following year found that there was no statistically significant difference in hippocampal subarea volume between the POE group and the epilepsy group, while the bilateral hippocampal fissure was larger in the POE group, and the difference was significant on the right side [55]. Although it is not exact whether there is a difference in hippocampal subregion volume between POE patients and epilepsy patients, in a study of hippocampal histopathology in patients with TLE (derived from surgery or autopsy), TLE patients with psychosis had less neuronal loss in the CA1 region and increased density of calcium-binding protein-positive neurons in the CA4 region compared with TLE patients without psychosis. This further indicates the possibility that different hippocampal subregions play variable roles in POE [56].

The alterations associated with epilepsy are not restricted to the medial temporal lobe. In fact, epilepsy is currently recognized as a brain network disorder [57]. Moreover, the network neuroscience concept can also be applied to psychiatric disorders [58], so brain network analysis of POE may also yield novel findings. A recent study found an overall thickening of the cortex in POE patients compared with epilepsy patients without psychosis, mainly concentrated in nodes of the cognitive control network and default mode network [59]. Noriaki Hirakawa compared the whole brain volumes of 14 IIP patients and 14 epilepsy patients and found that compared with epilepsy patients, the gray matter volumes of IIP patients in the left postcentral gyrus and the left supramarginal gyrus were significantly reduced [60]. This result suggests that gray matter volume reduction in the left postcentral gyrus and the left supramarginal gyrus may be a key region for IIP. In addition to gray matter, white matter may also have a role in the occurrence of POE. It has been found that TLE patients with interictal psychosis have significantly reduced fractional anisotropy in bilateral frontal and temporal white matter regions compared with those without psychosis [61]. A subsequent study performed diffusion tensor imaging (DTI) analysis of whole-brain white matter tracts and found that IIP patients showed more extensive and severe white matter damage than non-psychotic TLE patients, including anterior thalamic radiation, inferior fronto-occipital fasciculus, and inferior longitudinal fasciculus, and impaired network connectivity, mainly in the left limbic lobe and prefrontal cortex [62]. These findings suggest that white matter tract damage may play a role in the pathophysiological process of POE, but the mechanism needs to be further explored.

#### 4.1.2. Functional Neuroimaging

At present, there are few functional neuroimaging studies on POE, and most studies have used single-photon emission computed tomography (SPECT) to measure cerebral blood flow. A 1993 study that first used SPECT to compare regional cerebral blood flow (rCBF) between five POE patients and five epilepsy patients without psychotic symptoms found a significant reduction in rCBF index in the medial temporal region of the left hemisphere in POE patients [63]. Then, Mellers et al. found that blood perfusion in the left superior temporal gyrus was deficient in POE patients compared with controls (the epilepsy group and the schizophrenia group) when performing a verbal fluency task [64]. However, Ricardo et al. used SPECT to analyze rCBF in areas such as the frontal, temporal, and parietal cortex in 21 patients with POE and 23 patients with epilepsy and found no differences [65]. Therefore, it is controversial whether dysfunction in the temporal region of the dominant hemisphere is associated with the development of psychotic symptoms in patients with epilepsy. Most of the above studies were on IIP, while the results of studies on PIP were quite different from them, consistently showing hyperperfusion. One study examined rCBF in six PIP patients using SPECT and found reversible hyperperfusion abnormalities in the lateral temporal in PIP patients [66]. Leutmezer et al., in a study of five TLE patients with PIP, found that all patients showed bi-frontal and bi-temporal hyperperfusion during psychotic episodes compared to the non-psychotic state [67]. These results suggest that TLE with PIP may be associated with hyperactivation of temporal and frontal structures. Such hyperperfusion may reflect persistent subcortical discharge or simply a dysregulation of rCBF. 18F-fluorodeoxyglucose-positron emission tomography (18F-FDG PET) is a widely used imaging method for brain glucose metabolism. Compared to SPECT studies, the application of 18F-FDG PET in the investigation of patients with POE has been relatively restricted. Daichi Sone used 18F-FDG PET to detect patients with IIP and epilepsy. Hypometabolic brain regions from the ipsilateral temporal pole to the hippocampus were found in both groups. However, in voxel-level comparisons, 18F-FDG signals were significantly higher in the upper cerebellum, the superior cerebellar peduncle, and the midbrain in IIP patients [68]. These findings may reflect the involvement of the cerebellum in the underlying neurobiology of IIP. It is crucial to highlight that functional magnetic resonance imaging (fMRI) has found extensive application in the study of epilepsy and psychiatric disorders. However, research involving fMRI in the context of POE has been relatively sparse. fMRI offers the benefit of non-invasively evaluating dynamic brain activity. With technological advancements, future studies employing fMRI to investigate the mechanisms of POE could prove to be particularly significant (Table 2).

### 4.2. Neurobiology

At present, the neurobiological mechanism of POE remains unclear. The main research direction focuses on neurotransmitters and neuroinflammatory mediators, and other less-studied mechanisms include autoantibodies, parvalbumin, neurotrophic factors, paraoxonase-1, etc.

#### 4.2.1. Neurotransmitters

##### Glutamate

Glutamate is the main excitatory neurotransmitter in the brain, which acts on ionotropic and metabotropic receptors. Dysfunction in glutamate regulation may lead to network hyperactivity and neurotoxicity. Inotropic glutamate receptors include N-methyl-D-aspartate (NMDA), 2-carboxy-3-carboxymethyl-4-isopropenylpyrrolidine (kainate), and α-amino-3-hydroxy-5-methyl-4-isoazolepropionic acid (AMPA) receptors [69]. Metabolic glutamate receptors (mGluR) are a family of eight subtypes (mGluR1-8), which can be subdivided into three functional groups (I-III) based on receptor structure, signaling pathways, and physiological activity. Group I includes mGluR1/5, group II includes mGluR2/3, and group III includes mGluR4/6/7/8 [69]. NMDA receptors have long been of interest because NMDA receptor antagonists, such as ketamine, can induce psychotic-like symptoms [70]. A 2016 meta-analysis found that mRNA and protein levels of the NR1 subunit of the NMDA receptor were significantly lower in the prefrontal cortex of patients with schizophrenia than in healthy individuals [71]. The level of glutamate may also be related to schizophrenia. A meta-analysis conducted in 2023, encompassing 8256 individuals diagnosed with schizophrenia and 7532 healthy individuals, revealed significantly reduced levels of glutamate in the medial frontal cortex, elevated levels of glutamine in the thalamus, and heightened concentration of combined glutamate and glutamine in the basal ganglia among patients with schizophrenia, as opposed to their healthy counterparts [72]. Research pertaining to mGluRs is mainly preclinical, and various subunits in the three functional groups have not been associated with epilepsy and/or schizophrenia in the same way. Although there are almost no clinical studies on mGluRs, it cannot be denied that they are closely related to epilepsy and schizophrenia. If further clinical studies confirm the results of the current preclinical studies, it is promising to develop potential therapeutic targets for epilepsy/schizophrenia/POE. The following will review the role of different mGluRs in epilepsy and/or schizophrenia.

- mGluR1/5

A study has found that there are multiple non-synonymous single nucleotide polymorphisms (SNPs) associated with schizophrenia in the human gene encoding mGluR1 (GRM1). These SNPs were shown to be hereditary and not only associated with schizophrenia. The mutation has also been found in relatives with epilepsy, depression and anxiety, and drug and alcohol dependence [73]. Previous studies have shown that striatal dopaminergic hyperactivity is associated with schizophrenia [74,75]. Moreover, it has been found that the activation of mGluR1 can inhibit striatal dopamine release, and a novel selective mGluR1 positive allosteric modulator (PAM) exerts potent antipsychotic effects through an endocannabinoid-dependent mechanism [76]. A study showed reduced mGluR1 expression in the thalamic region, excluding the ventroposterolateral nucleus, in rats. Systemic treatment with SYN119, an mGluR1 enhancer, can reduce spontaneous spike discharges and spike discharges in epileptic rats. However, the noncompetitive mGluR1 antagonist JNJ16259685 increased the incidence of these discharges. These data suggest that the absence of epilepsy may be associated with a decrease in mGluR1 in the thalamus [77]. Therefore, compounds that enhance the activity of mGluR1 could potentially be developed into innovative treatments for the absence of epilepsy.

The gene encoding the mGluR5 (GRM5) is a promising target for the treatment of various symptoms of schizophrenia. Wang et al. reported a decrease in mGluR5 signaling in the dorsolateral prefrontal cortex of patients with schizophrenia postmortem. Additionally, the authors found that serine and tyrosine phosphorylation of mGluR5 was increased in the dorsolateral prefrontal cortex, which may contribute to reduced mGluR5 signaling due to receptor desensitization observed in schizophrenia [78]. In addition to this, lower mGluR5 availability in the left temporal cortex and caudate was associated with higher levels of negative symptoms, lower levels of global functioning, and worse cognitive task performance in male patients with chronic schizophrenia [79]. These findings support that dysregulation of mGluR5 signaling/function may underlie the pathophysiology of schizophrenia.

These studies suggest that mGluR1/5 reduction may play a common physiological role in the development of epilepsy and schizophrenia and that compounds that enhance mGluR1/5 activity may have the potential to be developed as novel anti-POE drugs.

- mGluR2/3

Genome-wide association studies have identified genetic variants in the gene coding for mGluR3 (GRM3) as a potential risk factor for the development of schizophrenia [80], but the genetic association analysis of the gene coding for mGluR2 (GRM2) and schizophrenia did not find any association [81]. Preliminary data from current experimental models of schizophrenia point to a decrease in mGluR2 in the prefrontal cortex, but conflicting results were found in TLE patients. In fact, mGluR2/3 distribution changed dynamically during epileptogenesis [82]. Previous studies have found that mGluR3 is involved in the regulation of cortico-thalamo-cortical circuits, and LY2794193, an mGluR3 PAM, may be one of the candidates for the treatment of absence epilepsy in rats [83]. Cameron et al. found that a PAM of mGluR2 enhanced the efficacy and potency of levetiracetam (LEV) [84] but not sodium valproate and lamotrigine [85] in a widely used preclinical model of refractory epilepsy. This synergistic effect appears to be unique to LEV. If further validated in future clinical studies, the pairing of LEV PAM with mGluR2 PAM could be a viable multi-drug therapy for patients with refractory epilepsy. In both neurological disorders characterized by dysfunctional glutamate neurotransmission, mGluR2/3 may play a crucial part as a protective negative feedback system and provide targets for novel therapies [82].

- mGluR4/7/8

The mGluR4-selective agonists LSP1-2111 [86] and LSP4-2022 [87] have been found to have antipsychotic-like effects. However, it has been found that mGluR4 activation is involved in the generation of absence seizures, which may be exacerbated by the use of positive allosteric modulators [88]. Genetic variants in the gene encoding the mGluR7 (GRM7) have also been shown to be associated with schizophrenia [89,90]. Similar to mGluR4, a series of studies have found that mGluR7 is also involved in the pathophysiology of absence epilepsy and could be targeted by therapeutic intervention [91]. A review points to the intriguing notion that altered mGluR7 function could lead to the pathological oscillations in the thalamo-cortical network underlying absence epilepsy and that drugs that selectively enhance mGluR7 function may be therapeutically useful in treating absence epilepsy [92]. At present, there are limited studies on mGluR4/7 in epilepsy and schizophrenia, and the mechanism of its opposite effects is not clear, which still needs further research and interpretation.

mGluR8 agonist (S)-3, 4-DCPG inhibits sound-induced seizures and pilocarpine-induced status epilepticus in mice [93]. Moreover, a study found that (S)-3, 4-DCPG inhibited Schaffer collateral-CA1 (SC-CA1) synaptic transmission in epileptic tissues by a presynaptic mechanism [94]. Furthermore, mGluR8 was demonstrated to be developmentally downregulated in the CA1 region, whereas tissue from chronically epileptic subjects exhibits significant transcriptional up-regulation of mGluR8, returning to levels observed in immature states. This is thought to be a compensatory response that counteracts the hyperexcited epileptic state and provides evidence to support the potential of mGluR8 agonists as useful anticonvulsants [94]. However, from the conclusions drawn by some of the existing studies, it is possible that the mGluR8 is not directly involved in the behaviors associated with schizophrenia [95,96]. Therefore, mGluR8 may not play an important role in the development of POE.

Although no studies have directly explored the role of mGluRs in POE animal models or patients, many studies have found that mGluRs were involved in the pathophysiology of epilepsy and schizophrenia, and different functional groups of receptors have different effects. To be specific, the downregulation of mGluR2/3/7/8 function may be involved in the occurrence of epilepsy, especially the occurrence of absence epilepsy. Several studies have suggested that mGluR5 dysfunction may be the pathophysiological basis of schizophrenia, while mGluR2/8 may not be closely related to schizophrenia. More importantly, mGluR1 activator has both antiepileptic and antipsychotic effects, suggesting that mGluR1 dysfunction may be one of the pathophysiological mechanisms of POE, but its exact mechanism still needs to be further explored in the future. In addition, we also found that mGluR4 has opposite effects on epilepsy and schizophrenia. This result does not show that the pathophysiological mechanism of epilepsy and schizophrenia is the opposite pathway but shows that the mechanism of these two diseases is very complex. There is still a long way to unlock its mystery. However, current studies on mGluRs are mostly limited to preclinical studies, and the animal model is mostly absent in epilepsy. This does not mean that absence epilepsy is more closely related to psychosis than other seizure types. In fact, clinical data show that TLE is closely related to psychosis. In the future, it is necessary to explore the relationship between mGluRs and psychotic disorders in other animal models of epilepsy, especially in TLE.

##### Gamma-Aminobutyric Acid (GABA)

Gamma-aminobutyric acid (GABA) is the most common inhibitory neurotransmitter in the human brain. One study showed that patients with schizophrenia had lower mRNA and protein levels of glutamate decarboxylase (GAD) 67 [97], which is an enzyme that synthesizes GABA, compared with healthy individuals [98]. A 2022 meta-analysis found lower GABA levels in the middle cingulate cortex of patients with FEP and in the occipital cortex of patients with schizophrenia compared with healthy people [99]. These findings could suggest that cortical GABA levels are lower in schizophrenia than in healthy individuals. Furthermore, research has indicated that untreated schizophrenic patients exhibit reduced levels of the α5 subunit of the GABA receptor in the hippocampus, as compared to healthy individuals [100]. Similar to schizophrenia, GABA is also closely related to epilepsy. Defects in GABAergic signaling have been proved in epilepsy patients; for example, patients with epilepsy have lower GABA levels than healthy patients, and patients with poor seizure control have lower brain GABA levels than those with good seizure control [101]. In animal models, rats lacking the GAD65 isoform show features of epilepsy and premature death [102]. Clinically, anti-GAD65 antibodies have been identified in acute and chronic epilepsy [103]. Additionally, epilepsy-associated mutations have been identified in many different GABAA receptor (GABAAR) subunits, such as α1, β3, γ2, and δ [104]. What is more, GABA antagonists have been found to induce seizures, while GABA agonists have anticonvulsant effects [105]. Clinically, benzodiazepines (e.g., diazepam) are the drug of choice for the treatment of status epilepticus [106]. All of these data emphasize the importance of GABA in the pathophysiology and treatment of epilepsy. Although current studies have been limited to investigating the role of GABA in epilepsy and schizophrenia alone, previous studies have found that GABAAR subunit expression changes, GABA binding, and lower GABA levels can be observed in patients with TLE and comorbid anxiety and/or depression [107]. Therefore, we might hazard a guess that GABA may also play a role in the pathophysiology of POE. Of course, future studies are needed to prove this hypothesis.

##### Dopamine

The dopamine hypothesis has been an important pathological theory for schizophrenia for decades. A meta-analysis showed the ability of striatal dopamine synthesis and release in schizophrenia patients is higher [108]. Similarly, ultra-high-risk (UHR) individuals for schizophrenia also exhibit higher striatal dopamine synthesis and release capacity compared with healthy controls [109,110,111]. These changes were more pronounced in individuals who subsequently developed psychosis [109]. Dopamine is also closely related to epilepsy. It has now been documented that dopaminergic D1/D2 injection into the dorsal striatum can inhibit seizures. In addition, combined injection of low doses of a D1 and a D2 agonist in the dorsal striatum had an additive effect in the absence of seizure suppression [112]. Using neuroimaging to examine dopamine receptors, D2/3 receptors were found to be significantly reduced in the anterior caudate-putamen of rats [113]. From the results of the above studies, we can see that the changes in dopamine levels in schizophrenia and epilepsy are opposite. This result leads one to wonder what happens to dopamine levels in the striatum of POE patients. Perhaps it is not constant and shows different levels of change at different stages of the disease. Further studies are expected to explain this interesting phenomenon in the future.

#### 4.2.2. Neuroinflammatory Mediators

Neuroinflammation is the response of the central nervous system to various injuries and is mainly associated with the activity of innate immune cells, such as microglia and astrocytes [114]. Both animal experiments and clinical studies have found that neuroinflammation has an undeniable impact on epilepsy and schizophrenia [115,116].

##### Interleukin-1β (IL-1β)

Interleukin-1β (IL-1β) is an important inflammatory mediator whose expression is maintained at a low basal level in most tissues under physiological conditions but can increase rapidly in response to various inflammatory reactions [117]. A meta-analysis found a significant increase in serum IL-1β levels in patients with chronic schizophrenia [118]. This IL-1β up-regulation is also manifested in FEP [119]. What is more, serum levels of IL-1β were significantly higher in UHR individuals for schizophrenia than in healthy individuals, suggesting that IL-1β may be a specific biomarker for UHR [120]. These results suggest that IL-1β may be involved in the process of immune inflammation before the onset of schizophrenia and will continue to affect the progression of the disease.

At present, studies have generally suggested the involvement of IL-1β in epilepsy, and most studies have shown that IL-1β levels in both serum and cerebrospinal fluid are significantly increased in patients with epilepsy [121,122]. Similarly, in an animal model of kainic acid-induced seizures, up-regulation of IL-1β mRNA was observed in several brain regions, including the hippocampus, cerebral cortex, striatum, thalamus, and hypothalamus [123]. In addition, administration of IL-1β to the hippocampus prior to kainic acid induction significantly increased the severity and duration of seizures in rodents and injection, while an IL-1β antagonist blocked this effect [124,125,126]. The above studies suggest that the up-regulation of IL-1β expression has an epileptogenic effect and is positively correlated with the severity and duration of seizures. However, a study of the role of intraventricular injection of IL-1β in epilepsy found that IL-1β had protective and antiepileptic effects [127]. Pascoal et al. also found that when animals were exposed to more IL-1β, there was better tolerance to the acute phase of the pilocarpine-induced epilepsy model, and mortality was reduced [128].

We hypothesized that the opposite results might be due to the regulation of IL-1β content in different parts of the brain, or it could be that IL-1β has a dual effect with dose. In any case, as an inflammatory factor that is important for both epilepsy and schizophrenia, although its role in epilepsy is controversial, we still believe that it is of great value to further investigate the role of IL-1β in the neuroimmune mechanism of POE.

##### Interleukin-6 (IL-6)

Interleukin-6 (IL-6) is a pleiotropic proinflammatory cytokine, and its dysregulation is associated with chronic inflammatory and autoimmune diseases [129]. It has been suggested that genetically determined IL-6 is associated with brain structure and may affect regions involved in developmental neuropsychiatric disorders, including schizophrenia and autism [130]. In patients with FEP who were not treated with APDs, serum IL-6 levels were significantly increased [131,132]. Elevated levels of IL-6 in the serum are associated with more severe negative symptoms in patients with schizophrenia and other psychoses [133]. Additionally, after 4 weeks of treatment for FEP, the level of IL-6 in serum decreased significantly (*p* = 0.012) [134]. Therefore, it seems that serum IL-6 level has great potential in predicting the severity of negative symptoms and the response to APDs.

In epilepsy patients, the serum IL-6 level during the interictal period was significantly higher than that in the normal population [122]. The level of IL-6 in serum and cerebrospinal fluid of patients with status epilepticus is significantly higher than that of patients without status epilepticus [135], suggesting that seizures may cause further accumulation of IL-6. Renli Qi et al. also found that the levels of IL-6 and IL-1β in the hippocampus were significantly increased in the epileptic rat model, and VNS could greatly reduce the levels of these inflammatory factors [136], indicating that VNS may play an antiepileptic effect by inhibiting the inflammatory process of epileptic foci. Another study showed that the IL-6 receptor antagonist tocilizumab was able to significantly reduce the occurrence of absence seizures in adult rats while at the same time reducing the occurrence of comorbid depressive-like behaviors [137]. This suggests that the IL-6 signaling transpathway may play a role in the pathway of comorbid depressive disorder in epilepsy. Whether this effect also occurs in other comorbidities of epilepsy, such as POE, is a promising study.

##### Tumor Necrosis Factor-α (TNF-α)

Tumor necrosis factor-α (TNF-α) is a proinflammatory cytokine that is also mainly released by activated microglia and astrocytes in response to neuroinflammatory challenges. In addition, TNF-α-mediated signal transduction can trigger microglia to release glutamate by up-regulating glutaminase, thereby causing excitatory neurotoxicity [138]. The serum level of TNF-α in patients with FEP was significantly higher than that in healthy controls [120,132]. In addition, it has also been found that faster conversion of UHR to FEP is associated with higher TNF-α [139]. Similar to IL-6, TNF-α is associated with more severe negative symptoms in schizophrenia and other psychotic disorders [131,133]. The serum levels of TNF-α in patients with acute relapse of chronic schizophrenia were significantly increased [140]. These results indicate that TNF-α, similar to IL-6, can be used as a predictor of the severity of negative symptoms of psychosis. In addition, the detection of TNF-α in UHR individuals helps to monitor and manage the process of transformation to FEP.

The serum levels of TNF-α in patients with epilepsy are higher than those in healthy people and are positively correlated with the severity of epilepsy [121]. A related study found that higher levels of IL-6 and TNF-α in the serum were closely related to increased susceptibility to epilepsy [141]. In addition, it has been shown that VU0360172, a mGlu5 PAM, can reduce seizures in virus-induced epilepsy model mice and also reduce IL-6 and TNF-α production [142]. The above studies suggest that in the occurrence of epilepsy, TNF-α and IL-6 are closely related to the severity of epilepsy, the increase in susceptibility, and the mGlu5-related pathway.

In conclusion, a large number of studies have shown that both epilepsy and schizophrenia are closely related to neuroinflammatory mediators. IL-1β, IL-6, and TNF-α, as important inflammatory factors in these two diseases, may play a role in the same or different pathways, affecting the generation, development, and prognosis of neuropsychiatric diseases. It is worth mentioning that recent studies on the microbiota–gut–brain axis have found that the disorder of gut microbiota may be related to the occurrence and progression of neuropsychiatric diseases, such as epilepsy, Parkinson’s disease, and schizophrenia [143]. Moreover, supplementation of probiotics significantly reduced the levels of TNF-α and IL-6 in the brains of rats [144]. This undoubtedly opens up a new idea in the direction of treatment.

#### 4.2.3. Others

##### Autoantibodies

For more than a decade, many scholars have paid much attention to the correlation between autoimmunity, epilepsy, and mental disorders. One study found that 11% of patients in two large epilepsy cohorts had antibodies against autoantigens, such as NMDA receptors, voltage-gated potassium channel complex protein, glycine receptors, and glutamic acid decarboxylase [145]. A systematic review encompassing 81 studies revealed that patients with schizophrenia exhibited significant elevations in positive titers of 20 different autoantibodies when compared to control subjects. Additionally, the prevalence of positive anticardiolipin IgG and NMDA receptor titers was significantly increased in first-episode psychotic patients. Schizophrenia patients also demonstrated significant increases in the absolute titers of anticardiolipin IgG, IgM, and nerve growth factor [146]. And one study has found associations between autoantibodies to six antigens and specific psychopathology symptoms: anti-AP3B2 (persecutive delusions), anti-TDO2 (hallucinations), anti-CRYGN (initial insomnia), anti-APMAP (loss of appetite), anti-OLFM1 (above-median cognitive function), and anti-WHAMMP3 (anhedonia and dysphoria) [147]. These studies add to the evidence that autoimmunity is closely related to epilepsy and psychosis. A previous study raised the hypothesis that PIP may be an autoimmune phenomenon mediated by autoantibodies against synaptic antigens. They proposed that prolonged or recurrent seizures lead to transient blood–brain barrier dysfunction, during which the brain is exposed to pathogenic autoantibodies. They emphasized that certain characteristics of PIP can be elucidated by this mechanism, such as the lucid interval between seizures and psychotic episodes, as well as the progression of psychosis to chronic interictal periods in some cases [148]. However, this hypothesis cannot explain why many of the psychotic symptoms of PIP patients resolve spontaneously within a few days or weeks. At present, the autoantibody research on POE is still limited, and more research is still needed in the future.

##### Parvalbumin

Parvalbumin belongs to a family of calcium-binding proteins, and it is predominantly found within subpopulations of inhibitory GABAergic interneurons across various brain regions, such as the neocortex, cerebellum, hippocampus, and the reticular nucleus of the thalamus [149]. Thus, it is possible that the absence of parvalbumin implies a loss of subpopulations of inhibitory neurons, which may induce seizures. One study found that GABAergic interneurons expressing parvalbumin mediate the antiepileptic effects of neuregulin 1 (NRG1) -ErbB4 signaling [150]. In addition, a study has shown that the inhibition of retrosplenial cortex parvalbumin interneurons leads to dissociation-related behaviors [151]. This suggests that the absence of parvalbumin may be involved in the development of epilepsy and psychosis. Nevertheless, several studies have proposed that the observed reduction in parvalbumin immunoreactivity may be related to the level of parvalbumin protein expression rather than to the loss of GABAergic neurons expressing parvalbumin [152,153]. Precisely because parvalbumin loss does not inherently indicate a reduction in subsets of GABAergic neurons, we cannot directly link parvalbumin loss to epilepsy and schizophrenia. It probably still needs to act on GABAergic pathways to play a role in these two diseases.

##### Neurotrophins (NTs)

Some scholars have also found that there are overall structural abnormalities and changes in the expression of NTs and their receptors in the hippocampus of epilepsy patients, and they are differentially regulated in patients with POE [154]. However, the outcomes of various studies are inconsistent in the levels of brain-derived neurotrophic factor and tyrosine kinase receptor, with some indicating no change, others showing a decrease, and still others demonstrating an increase [155,156,157]. The clinical significance of these results remains to be determined, but the function of NTs and their receptors is to be able to modulate central nervous transmission and promote neuroplasticity, so further studies are undoubtedly valuable for the development of new therapeutic options.

##### Paraoxonase 1 (PON1)

Research has revealed that oxidative stress toxicity and diminished antioxidant defense mechanisms are pivotal in the pathophysiology and associated psychopathology of TLE and medial temporal sclerosis (MTS) [158]. Subsequently, a study in 2022 found that the decrease in PON1 activity would participate in the occurrence of psychotic disorders (including psychosis, anxiety, depression, etc.) by increasing oxidative stress in patients with TLE and MTS comorbid with mental disorders [159]. This mechanism partly elucidates the significant comorbidity observed between TLE and psychiatric symptoms, indicating that the activity of the PON1 enzyme could serve as a potential therapeutic target for managing seizures and psychiatric comorbidities in individuals with TLE (Table 3).

### 4.3. Genetics

In recent years, studies on gene sequencing of patients with epilepsy [160] and psychotic disorders [161] have attracted increasing attention. Although there is no large-scale gene sequencing study for patients with POE, there are some genetic variants that show the phenotypes of epilepsy and psychosis at the same time. These genetic variants may be the common causes of the two diseases, but further studies on the specific mechanisms are needed to verify.

#### 4.3.1. Single Gene Mutation

Currently, many studies have reported the occurrence of POE caused by a single gene mutation. Adenosine kinase (ADK) is an enzyme that catalyzes the transfer of γ-phosphate from adenosine triphosphate to adenosine, which interacts with dopamine and glutamate neurotransmitters. Thus, changes in *ADK* may also be associated with schizophrenia and/or epilepsy. Hiroki Kimura et al. reported a case presenting with POE and found a loss of function variant in the *ADK* region. A subsequent search for the breakpoint revealed that six exons (from exon 3 to exon 8) had been deleted and that this deletion was the result of a loss (frameshift) of the functional variant [162]. This undoubtedly suggests to us that the copy number variation in the *ADK* region may be closely related to POE. The genes that have been reported to be associated with POE due to copy number variations also include deletion of *CNTN6* [163] and haploinsufficiency of *CNTNAP2* [164]. Familial inherited disorders also deserve attention. *PCDH19*-GCE refers to cluster epilepsy in girls caused by pathogenic variants in the protocadherin 19 gene. Some scholars have found that one-fifth of *PCDH19*-GCE patients develop schizophrenia and other mental disorders in the late stage [165]. Therefore, the monitoring, identification, and appropriate treatment of psychotic symptoms in women with *PCDH19*-GCE should perhaps be part of routine care in this population.

Additionally, certain studies have identified potential shared genetic markers between epilepsy and depression, though they have yet to establish a definitive link between a specific gene and POE. For instance, reduced levels of *BDNF* and increased methylation of the *SLC6A4* gene promoter have been notably correlated with the occurrence of major depression or a history of depression in patients with TLE [166]. *BDNF* Met allele carriers exhibited more severe depressive symptoms on the Personality Assessment Scale compared to individuals lacking the Met allele (*p* = 0.004) [167]. It would be beneficial to further explore the role of these comorbid genes in more epilepsy comorbidities, especially in POE, which requires larger-scale studies.

#### 4.3.2. Chromosomal Segment Deletions

In addition to the possible effect of single-gene mutations on POE, there are some studies on the effect of chromosomal segment deletions on POE. In 2016, Forsingdal, A. et al. constructed a 15q13.3 knockout mouse model and found that 15q13.3 deletion mice were more likely to develop epilepsy, autism, and schizophrenia than wild-type mice. Moreover, the physiological behavior disorder of 15q13.3 homozygous deletion mice is more obvious than that of hemizygous deletion mice [168], which indicates gene dose dependence, contributes to the understanding of the biological mechanism of severe mental disorders, and also provides a method for studying POE animal models. A Danish study included 12 patients with a phenotype of 17q12 deletion and 26 patients with a phenotype of 17q12 duplication. They found that a range of symptoms, including epilepsy and schizophrenia, were seen in both groups [169], which suggested that some people may be sensitive to the dose changes in genes located in the chromosome 17q12 region. A variable phenotype of epilepsy, intellectual disability, and schizophrenia caused by a 12p13.33-p13.32 terminal microdeletion was identified in a Korean family. Among the six candidate genes in this region, *CACNA1C* may be the best candidate. However, its functional role in neurotransmission and its etiologic association with neuropsychiatric disability remain unclear, and further detailed molecular analysis of *CACN1C* transcripts is needed [170]. What is more, some scholars have found that patients with 22q11.2 deletion syndrome may have psychotic disorders, myoclonic epilepsy, and cognitive impairment at the same time [171]. APDs for patients with 22q11.2 deletion syndrome often require caution because of increased side effects, including seizures. Clozapine may be a relatively safe and effective option [172]. Although most of the above studies were small, they have at least been able to further clarify the association between epilepsy and psychosis at the genetic level and have narrowed the range of candidate genes to some extent.

#### 4.3.3. Genetic Polymorphisms

In 2014, Jose Augusto Bragatti et al. initiated the first study on the association between *tryptophan hydroxylase 2* (*TPH2*) gene polymorphisms and psychiatric comorbidities in epilepsy [173]. They found that the presence of the T allele in the rs4570625 polymorphism was associated with psychosis. Although they were unable to observe a further association, it still suggests a possible influence of serotonergic allele variants on epilepsy with psychotic disorders. It has also been reported that patients with refractory epilepsy who carried the *catechol-O-methyltransferase* (*COMT*) Val (rs4680) allele had more severe symptoms of schizophrenia (*p* = 0.007) on the personality assessment inventory than non-carriers [167]. This suggests that *COMT* may play an important role in the pathophysiology of patients with psychosis or intractable epilepsy. However, this study was limited to patients with intractable epilepsy from a single center, and the results may not be generalizable to a larger epilepsy population, and further studies are needed. Psychosis, hyperhomocysteinemia, and multivitamin deficiency have been reported in *methylene tetrahydrofolate reductase* (*MTHFR*) C677T homozygotes with epilepsy. They believe that psychotic symptoms are caused by vitamin deficiency after taking ASMs and can be treated by changing ASMs as well as different vitamins [174] (Table 4).

## 5. Conclusions and Prospects

Psychosis is a prevalent comorbidity among patients with epilepsy, significantly diminishing their quality of life. Clinically, the early identification of psychotic symptoms in patients with POE is crucial, as it allows for the timely administration of appropriate antipsychotic treatments and the selection of suitable ASMs, potentially improving patient prognosis. Consequently, it is imperative to closely monitor the emergence of psychotic symptoms in epilepsy patients, particularly those with a history of psychiatric disorders and those recently initiated on novel ASMs. Although numerous scholars posit that epilepsy surgery positively impacts the quality of life for POE patients and can be conducted under stringent psychiatric supervision, the effect of surgery on psychotic symptoms in POE patients remains ambiguous. Current research is limited by inadequate sample sizes and insufficient follow-up durations. Future clinical studies with larger sample sizes and extended postoperative follow-up periods are anticipated to elucidate whether the benefits of surgery outweigh the drawbacks for POE patients.

Neuroimaging research on POE has indicated potential roles for alterations in hippocampal/amygdala volume, hippocampal subregion volume, gray matter volume, white matter tracts, and rCBF in POE. However, the findings have been inconsistent. We suspect that this lack of reproducibility could be attributed to inadequate sample sizes and confounding factors such as drugs. Furthermore, the overwhelming majority of research has been confined to IIP and PIP, and the studies on other POE types (such as FN) are devoid. We think that this is not because other types of POE imaging studies are less valuable but mainly because IIP and PIP patients are more common and easier to recognize in clinical practice than others. Additionally, we hold the view that, given its nature as a brain network disorder, the investigation of POE should extend beyond isolated brain regions and instead encompass the entire brain network. With the advancement of imaging technology, future research on the functional connectivity of brain networks in POE is expected to be further deepened, and we believe that some interesting findings can be found in this field.

Neurotransmitters have been the focal point of research into the pathophysiological mechanisms of epilepsy and schizophrenia. In mGluRs, it has been observed that the activation of mGluR1 exhibits both antiepileptic and antipsychotic properties. Nevertheless, there is a lack of direct research on mGluR1 in POE. It is reasonable to conjecture that a pathway related to mGluR1 may be one of the mechanisms of POE, and we hope that future studies will test this. In contrast, the activation of mGluR4 has opposite effects in epilepsy and psychosis, which suggests the possibility of multiple pathways and mechanisms in the development of epilepsy and psychosis and gives us a further understanding of the complex bidirectional interaction between them. In addition, a large number of studies have shown that both epilepsy and schizophrenia are closely related to neuroinflammatory mediators. IL-1β, IL-6, and TNF-α, as important inflammatory factors in these two diseases, may function in the same or different pathways. In recent years, the proposed microbiota–gut–brain axis has opened up a new idea for the treatment of POE. Furthermore, the preliminary explorations of autoantibodies, parvalbumin, NTs, and PON1 in POE make us no longer limited to the study of neurotransmitters or neuroinflammatory mediators and provide other good entry points for the study of POE mechanisms.

The influence of genetic factors on epilepsy and psychosis is undeniable. We review some of the single-gene mutations, chromosome fragment deletions, and genetic polymorphisms that show POE phenotypes. However, these studies have not been further explored at the transcriptional and translational levels, and the downstream pathways of these mutations remain unclear. We consider that it is necessary to perform whole-exome or even whole-genome sequencing in a larger POE population in the future and further functional validation of suspected mutations to find effective therapeutic targets at different levels. In addition, if the phenotype can be linked to the molecular level, it would be very meaningful in clinical diagnosis and treatment.

## Figures and Tables

**Table 1:** Clinical classification of POE.

Clinical Classification	PrP	IP	IIP	PIP	FN
Temporal relationship with epilepsy	A few hours (rarely up to 3 days) before the onset of seizures.	At the same time (a manifestation of seizures)	After the seizures, during the interictal period and no clear time relationship	A few hours to a few days after the seizures, generally no more than 1 week	At least 1 week after complete cessation of clinical seizures and/or reduced EEG seizure activity
Clinical feature	(a) Loss of reality (b) Depersonalization (c) Obsessive thinking (d) Premonitory signs of an ideological movement (e) Hallucination, anxiety, euphoria	(a) Thoughts of persecution (b) Anxiety/fear (c) Negativity (d) Irritability, aggression (e) Most common in TLE	(a) More conceptual disturbances (such as tautology, digress, loose association) (b) More negative symptoms (such as emotional dullness or withdrawal, lack of interest)	(a) Prominent emotionally charged delusions and hallucinations (b) Violent and/or suicidal behavior (c) A lucid interval (d) Common in TLE	(a) Thought disorders (b) Delusions (c) Hallucinations (d) Marked mood changes (e) Hypomania or depression (f) Anxiety with depersonalization and derealization
Treatment	No need for APDs	No need for APDs	Early use of APDs may shorten the duration of psychotic symptoms (selected based on efficacy and tolerability)	Generally, no need for APDs; benzodiazepines alone or in combination with APDs for frequent episodes	Cease of the ASMs (ASMs trigger) or use of APDs (other triggers)
Prognosis	Disappear with the seizures	Disappear with the end of seizures	Subside in months or years	Usually subside spontaneously within a few days or weeks or transform into IIP	Unknown

PrP: preictal psychosis of epilepsy; IP: ictal psychosis of epilepsy; IIP: interictal psychosis of epilepsy; PIP: postictal psychosis of epilepsy; FN: forced normalization; APDs: antipsychotic drugs; TLE: temporal lobe epilepsy; EEG: electroencephalogram; ASMs: anti-seizure medications.

**Table 2:** Summary of neuroimaging findings on POE.

Neuroimaging	Consequences	Researches
Structural neuroimaging	Reduced bilateral hippocampal and amygdala volume	Maier, M. et al., 2000 [49]
	No difference in total hippocampal volume	Briellmann, R.S. et al., 2000 Tebartz Van Elst, L. et al., 2002 [50,51]
	Reduced left hippocampal volume	Marchetti, R.L. et al., 2003 [52]
	Increased bilateral amygdala volume	Tebartz Van Elst, L. et al., 2002 [51]
	Reduced bilateral posterior hippocampus volume	Allebone, J et al., 2019 [54]
	No difference in hippocampal subarea volume; increased bilateral hippocampal fissure	Allebone, J et al., 2020 [55]
	Overall thickening of the cortex	Allebone, J et al., 2022 [59]
	Reduced gray matter volume in the left postcentral gyrus and the left supramarginal gyrus	Hirakawa, N. et al., 2020 [60]
	White matter damage	Flügel, D. et al., 2006 Sone, D. et al., 2020 [61,62]
Functional neuroimaging	Hypoperfusion in the left temporal region in IIP	Marshall, E.J. et al, 1993 Mellers, J.D. et al, 1998 [63,64]
	No difference in rCBF in frontal, temporal and parietal regions in IIP	Guarnieri, R. et al, 2005 [65]
	Reversible hyperperfusion in the lateral temporal neocortex in PIP	Fong, G.C.Y. et al, 2002 [66]
	Hyperperfusion in bi-frontal and bi-temporal in PIP	Leutmezer, F. et al, 2003 [67]
	Hypermetabolism around the upper cerebellum in IIP	Sone, D. et al, 2022 [68]

IIP, interictal psychosis of epilepsy; PIP, postictal psychosis of epilepsy; rCBF, regional cerebral blood flow.

**Table 3:** Summary of neurobiology findings on POE.

Biomarker	Possible Function After Activation
Neurotransmitters	
mGluRs	
mGluR1	Antiepileptic/Antipsychotic
mGluR2/3/7/8	Antiepileptic
mGluR5	Antipsychotic
mGluR4	Inducing epilepsy/Antipsychotic
GABA	Antiepileptic/Antipsychotic
Dopamine	Antiepileptic/Inducing psychosis
Neuroinflammatory mediators	
IL-1β	Inducing epilepsy or antiepileptic/Inducing psychosis
IL-6	Inducing epilepsy/Inducing psychosis
TNF-α	Inducing epilepsy/Inducing psychosis
Others	
Autoantibodies	Inducing epilepsy/Inducing psychosis
Parvalbumin	Obscure
NTs	Obscure
PON1	Antiepileptic/Antipsychotic

mGluR, metabolic glutamate receptors; GABA, gamma-aminobutyric acid; NTs, neurotrophins; PON1, paraoxonase 1; IL-1β, interleukin-1β; IL-6, interleukin-6; TNF-α, tumor necrosis factor-α.

**Table 4:** Summary of genetics findings on POE.

Biomarker	Possible Mutation in POE
Single gene mutation	
*ADK*	Deletion
*CNTN6*	Deletion
*CNTNAP2*	Haploinsufficiency
*PCDH19*-GCE	Missense/Deletion
Chromosomal segment deletions	
15q13.3	Deletion
17q12	Deletion/Duplication
12p13.33-p13.32	Deletion
22q11.2	Deletion
Genetic polymorphisms	
*TPH2*	rs4570625
*COMT*	rs4680
*MTHFR*	C677T

ADK, adenosine kinase; TPH2, tryptophan hydroxylase 2; COMT, catechol-O-methyltransferase; MTHFR, methylene tetrahydrofolate reductase.

## Data Availability

No new data were created in this manuscript.

## Abbreviations

POE, psychosis of epilepsy; DSM-V, Diagnostic and Statistical Manual of Mental Disorders (Fifth Edition); TLE, temporal lobe epilepsy; PrP, preictal psychosis of epilepsy; EEG, electroencephalogram; IP, ictal psychosis of epilepsy; APDs, antipsychotic drugs; IIP, interictal psychosis of epilepsy; FEP, first-episode psychosis; PIP, postictal psychosis of epilepsy; FN, forced normalization; ASMs, anti-seizure medications; DTI, diffusion tensor imaging; SPECT, single-photon emission computed tomography; rCBF, regional cerebral blood flow; 18F-FDG PET, 18F-fluorodeoxyglucose-positron emission tomography; fMRI, functional magnetic resonance imaging; NMDA, N-methyl-D-aspartate; AMPA, α-amino-3-hydroxy-5-methyl-4-isoazolepropionic acid; mGluR, metabolic glutamate receptors; SNPs, single nucleotide polymorphisms; GRM, gene encoding mGluR; PAM, positive allosteric modulator; LEV, levetiracetam; SC-CA1, Schaffer collateral-CA1; GABA, gamma-aminobutyric acid; GAD, glutamate decarboxylase; GABAAR, GABAA receptors; UHR, ultra-high-risk; IL-1β, interleukin-1β; IL-6, interleukin-6; TNF-α, tumor necrosis factor-α; NRG1, neuregulin 1; NTs, neurotrophins; PON1, paraoxonase 1; MTS, medial temporal sclerosis; ADK, adenosine kinase; TPH2, tryptophan hydroxylase 2; COMT, catechol-O-methyltransferase; MTHFR, methylene tetrahydrofolate reductase.
